# Parkinsonism in Gerstmann-Sträussler-Scheinker disease: A case report

**DOI:** 10.1016/j.ensci.2025.100587

**Published:** 2025-09-05

**Authors:** Santiago Poveda, Juan Sebastián Montealegre-Claros, Lina María Lancheros, Maria Alejandra Cruz, Oscar Bernal Pacheco

**Affiliations:** a Instituto Roosevelt, Bogotá, D.C., Colombia; b Hospital Militar Central, Bogotá, D.C., Colombia; c Hospital Universitario San Ignacio, Hospital Universitario Clínica San Rafael, Bogotá, D.C., Colombia

**Keywords:** Gerstmann-Sträussler-Scheinker disease, Spongiform encephalopathies, Prion diseases, Parkinsonism, PRNP mutation

## Abstract

**Background:**

Autosomal dominant prion diseases of the central nervous system, including Gerstmann-Sträussler-Scheinker disease (GSS), Creutzfeldt-Jakob disease, and fatal familial insomnia, are caused by mutations in the PRNP gene. These conditions exhibit highly variable clinical and pathological features, making diagnosis challenging, with poor survival outcomes. In Colombia, the incidence of prion diseases remains unknown. We report a case of GSS with parkinsonism, a rare presentation, emphasizing intrafamilial variability with the same pathogenic variant, underscoring the importance of reporting each case.

**Case presentation:**

A 55-year-old woman from Colombia presented with symptoms of instability, rigidity, and bradykinesia. Over one year, her condition progressed to cognitive decline, dysphagia, and severe motor impairment. Differential diagnostic studies were conducted. A pathogenic P102L mutation in the PRNP gene was identified, confirming an autosomal dominant inheritance pattern. Symptomatic management and interdisciplinary rehabilitation were initiated. This mutation led to the diagnosis of at least 10 symptomatic family members and allowed for genetic counseling of asymptomatic relatives.

**Conclusions:**

This case of GSS with the P102L mutation demonstrates a late onset and rapid progression with atypical parkinsonism presentation, without ataxia, which is predominantly reported in previous cases. The familial segregation of this mutation highlights the importance of monitoring and following at-risk relatives. While the disease remains incurable, knowledge of genetic predisposition allows for better family planning and appropriate medical support, improving quality of life and emotional support.

## Highlights

• Underdiagnosis of GSS persists in Latin America due to limited genetic access.
• GSS P102L case with parkinsonism as initial symptom and absence of ataxia.
• Atypical phenotype supports need to consider GSS in early parkinsonism.
• This case expands the phenotypic spectrum of PRNP P102L-associated GSS.
• Homogeneous parkinsonian phenotype observed across multiple family members.

## 1 Introduction

**Fig. 1:**
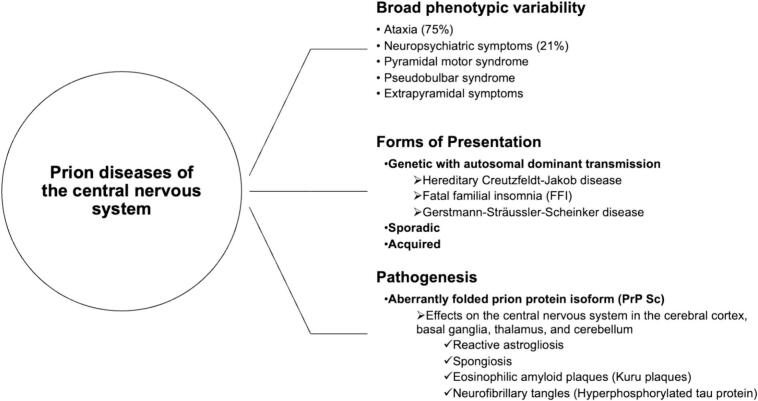
Overview of prion diseases of the central nervous system (1).

Spongiform encephalopathies, known as prion diseases, are late-onset neurodegenerative disorders with phenotypically variable presentations. Gerstmann-Sträussler-Scheinker disease (GSS) represents the prion disease with the greatest phenotypic and neuropathological variability (Fig. 1). To date, no effective treatment has been found, and the prognosis is fatal (1).

This case is significant due to the unusual presentation of parkinsonism in a patient with GSS, affected by a variant typically associated with ataxia. In Colombia, the underreporting of prion diseases limits the understanding of their incidence and clinical variability, highlighting the need for improved surveillance and diagnosis of these conditions.

## 2 Case presentation

The patient was a 55-year-old Colombian woman who initially presented with symptoms of gait instability, multiple falls, rigidity in all four limbs predominantly on the right side, generalized bradykinesia, resting and intention tremor in the upper limbs, flaccid dysarthria, and dysphagia initially for liquids, associated with irritability, insomnia, constipation, without oculomotor involvement, pyramidal signs, or ataxia. Her functional decline was progressive, and within one year, she became bedridden with cognitive impairment, motor aphasia, emotional lability, fecal and urinary incontinence, requiring a gastrostomy tube. She was treated with levodopa/carbidopa (750 mg/day) and clonazepam (2 mg/day), which provided partial relief of motor symptoms, anxiety, and sleep without adverse drug reactions. The patient received palliative care during her end-of-life process and died two years after the disease onset due to pneumonia.

**Fig. 2:**
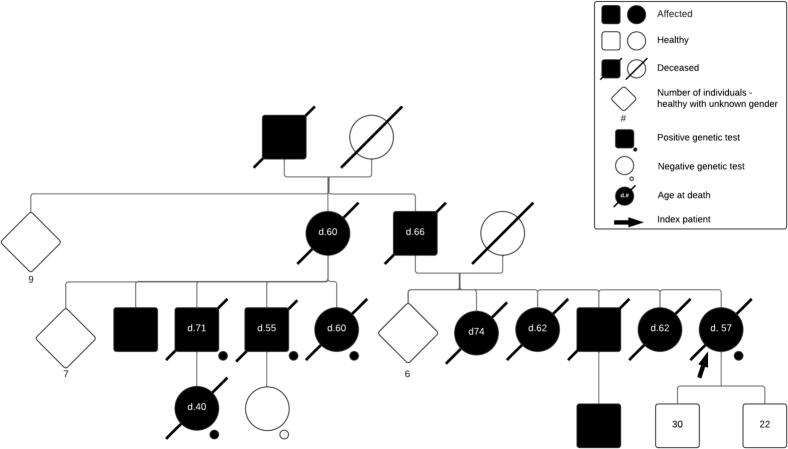
Family pedigree.

Family history revealed multiple paternal relatives with parkinsonism, dysarthria, and rapidly progressive cognitive decline, with death occurring a few years after symptom onset (Fig. 2). While clinical heterogeneity was broadly described among relatives, with predominant parkinsonian features, detailed characterization was limited due to the advanced stage or death of many relatives, and the fact that most lived in rural areas with scarce access to medical documentation. Nonetheless, at least 13 individuals were reported with neurological symptoms compatible with GSS.

A lumbar puncture was performed during the diagnostic workup to exclude infectious etiologies; however, specific prion protein markers in CSF (such as 14–3-3 protein) were not tested due to their high cost and the already high clinical suspicion based on the familial pattern. Differential diagnostic evaluation for extrapyramidal syndrome was conducted, including extensive laboratory tests (deficiency profiles; autoimmune, paraneoplastic, and toxic screens), electromyography, and nerve conduction studies, all of which were negative. Brain MRI revealed moderate T2 subcortical white matter hyperintensities, without cerebellar atrophy, diffusion abnormalities, or basal ganglia changes. A DAT scan was not performed due to limited access and cost.

Whole-genome sequencing revealed a heterozygous pathogenic variant, c.305C > T; p.Pro102Leu, in the PRNP gene, which is consistent with Gerstmann-Sträussler-Scheinker disease. This finding allowed the diagnosis of 13 family members and confirmed genetic testing in four of them.

## 3 Discussion

Prion diseases are neurodegenerative conditions caused by the aberrantly folded isoform of prion protein (PrP Sc), with polymorphisms in the PRNP gene (1,4,10). These mutations can be sporadic, acquired through contaminated food, human growth hormone administration, or surgical procedures, or genetic, as seen in Gerstmann-Sträussler-Scheinker disease (GSS), hereditary Creutzfeldt-Jakob disease, and fatal familial insomnia (1). GSS has an incidence of 1 to 10 cases per 100 million people per year, with unknown statistics in Colombia (2).

At least 16 prion protein gene mutations have been identified in GSS (2). The most common pathogenic mutation in PRNP is P102L, the first to be associated with the disease (1,4). The second most frequent is A117V (1). Other variants, described in more than 50 families with GSS, include P105L, Q160X, F198S, Q217R, Y218N, Y226X, D202N, and Q227X (6).

GSS encompasses the most diverse clinical and neuropathological spectrum (Table 1). Intrafamilial variability has been reported even with the same pathogenic variant, emphasizing the importance of reporting each case. Factors such as codon 129 polymorphism in PRNP, the presence of PrPSc isoforms, and co-pathologies (e.g., tauopathy) may explain this heterogeneity (1,4). Although the most common clinical presentation of GSS with the P102L mutation is ataxia (75,6 %), the prominent early extrapyramidal syndrome in this case and family is notable. Parkinsonism symptoms have been more frequently reported with the D202N variant (9). While this family showed phenotypic homogeneity toward early parkinsonism, the limited clinical details due to rural residence and patient death constrain further characterization. Up to 16.5 % of cases may have no family history (10). Disease progression also varies considerably, with an average survival of five years (2,4); in this case, the patient survived two years from symptom onset.

**Table 1:** GSS Phenotypes (1,2,6,7,10).

Clinical Phenotypes
Age of onset: 40–60 years
● Slowly progressive cerebellar ataxia followed by dementia ● Supranuclear palsy (less frequent) ● Eye movement disorders: gaze-evoked nystagmus, rebound nystagmus, impaired smooth pursuit, and hypometric saccades ● Parkinsonism ● Isolated dementia without ataxic syndrome, with behavioral changes (mood swings, aggressive behavior, paranoia) ● Pyramidal motor syndrome, spastic paraparesis ● Myoclonus and athetosis ● Blindness ● Severe sensorineural hearing loss ● Insomnia and excessive daytime sleepiness, with disorganized sleep architecture, obstructive sleep apnea (OSA), stridor, and REM sleep behavior disorder

Brain MRI is often normal early in the disease course, later showing cerebellar atrophy (42.9 %) and T2 hyperintensities in the corticospinal tract (20.6 %), putamen, and caudate nucleus (3,5,10). In our case, no significant imaging findings were documented on brain MRI. PET-SCAN imaging has shown decreased glucose uptake in the frontal and parietal lobes (superior parietal lobes, precuneus) (7). PET with [11C]PiB, which targets PrP-amyloid, is not useful for the in vivo evaluation of PrP-amyloid plaques in GSS patients (8). A DAT scan, although not performed in our patient, can demonstrate nigrostriatal degeneration, and may serve as a complementary tool in future evaluations of parkinsonian symptoms in GSS. (11)

The most characteristic pathological feature is the presence of multicentric eosinophilic amyloid plaques (Kuru plaques) derived from abnormal PrP products in the cerebral cortex, basal ganglia, and cerebellum (1,7,10). Additionally, neurofibrillary tangles formed by hyperphosphorylated tau protein have been observed (1). From a molecular standpoint, the PRNP gene encodes the cellular prion protein, which plays roles in synaptic function, neuroprotection, copper binding, and regulation of oxidative stress. Loss-of-function mutations like P102L lead to misfolding into the pathogenic isoform PrP Sc, which aggregates and disrupts neuronal function, contributing to the widespread neurodegeneration observed in prion diseases. This dysfunction correlates with motor, cognitive, and psychiatric manifestations (12). Post-mortem pathological study was not conducted for this patient. To date, no correlation has been established between pathological distribution and the different clinical manifestations (1). The presence of 14–3-3 protein in cerebrospinal fluid and the appearance of periodic synchronous discharges are uncommon in GSS patients and may lead to misdiagnosis of Creutzfeldt–Jakob disease (4).

## 4 Conclusions

This case of Gerstmann-Sträussler-Scheinker disease, caused by the P102L mutation in the PRNP gene, is characterized by an atypical presentation of parkinsonism from the initial stages without ataxia, which is uncommon in most reported cases with this mutation. This finding underscores the broad phenotypic variability of GSS, even within the same genetic mutation. In Colombia, as in other countries, the incidence remains unknown due to underreporting and the lack of adequate genetic diagnosis.

Since GSS can mimic other neurodegenerative disorders, this case report reinforces the need to include prion diseases in the differential diagnosis of extrapyramidal manifestations, through a comprehensive diagnostic approach. GSS should be suspected in patients with rapidly progressive neurodegenerative symptoms combining motor and cognitive signs, a family history, and a lack of sustained response to standard therapies. The disease has a poor prognosis with only symptomatic treatment, highlighting the need for a multidisciplinary approach in palliative care and genetic counseling.

## 5 Limitations

This case report has several limitations inherent to single-case studies. First, neuropathological confirmation was not available, preventing direct correlation of clinical and genetic findings with the characteristic histopathological distribution of Gerstmann-Sträussler-Scheinker disease. Second, cerebrospinal fluid biomarkers of prion disease (such as 14–3-3 protein or RT-QuIC) and advanced functional neuroimaging techniques (DAT-SCAN, PET-SCAN) were not performed, limiting further characterization of nigrostriatal involvement and differential diagnosis. Third, clinical information from affected relatives was restricted due to limited medical documentation and access to healthcare in rural areas, reducing the accuracy of intrafamilial variability analysis. Finally, as a single case report, the findings cannot be generalized and should be interpreted with caution, highlighting the need for larger case series or multicenter studies.

## CRediT authorship contribution statement

**Santiago Poveda:** Writing – review & editing, Writing – original draft, Methodology, Investigation, Conceptualization. **Juan Sebastián Montealegre-Claros:** Methodology, Investigation, Data curation. **Lina María Lancheros:** Writing – review & editing, Formal analysis, Conceptualization. **Maria Alejandra Cruz:** Writing – review & editing, Formal analysis. **Oscar Bernal Pacheco:** Writing – review & editing, Validation, Supervision, Conceptualization.

## Declaration of competing interest

None.

## References

[1] Eraña H., San Millán B., Díaz-Domínguez C.M. (2022). Description of the first Spanish case of Gerstmann–Sträussler–Scheinker disease with A117V variant: clinical, histopathological and biochemical characterization. J. Neurol..

[2] Ufkes N.A., Woodard C., Dale M.L. (2019). A case of Gerstmann-Straussler-Scheinker (GSS) disease with supranuclear gaze palsy. J. Clin. Mov. Disord..

[3] Shin M., Kim D., Heo Y.J., Baek J.W., Yun S., Jeong H.W. (2023). Gerstmann-Sträussler-Scheinker disease: a case report. J. Korean Soc. Radiol..

[4] Smid J., Neto Studart A., Landemberger M.C., Machado C.F., Nóbrega P.R., Canedo N.H.S., Schultz R.R., Naslavsky M.S., Rosemberg S., Kok F., Chimelli L., Martins V.R., Nitrini R. (2017). High phenotypic variability in Gerstmann-Sträussler-Scheinker disease. Arq. Neuropsiquiatr..

[5] Tesar A., Matej R., Kukal J., Johanidesova S., Rektorova I., Vyhnalek M., Keller J., Eliasova I., Parobkova E., Smetakova M., Musova Z., Rusina R. (2019). Clinical variability in P102L Gerstmann-Sträussler-Scheinker syndrome. Ann. Neurol..

[6] Chen Z., Guo J., Ran N., Zhong Y., Yang F., Sun H. (2023). A family with mental disorder as the first symptom finally confirmed with Gerstmann-Sträussler-Scheinker disease with P102L mutation in PRNP gene - case report. Prion.

[7] Yoshimura M., Yuan J.H., Higashi K., Yoshimura A., Arata H., Okubo R., Nakabeppu Y., Yoshiura T., Takashima H. (2018). Correlation between clinical and radiologic features of patients with Gerstmann-Sträussler-Scheinker syndrome (Pro102Leu). J. Neurol. Sci..

[8] Deters K.D., Risacher S.L., Yoder K.K., Oblak A.L., Unverzagt F.W., Murrell J.R., Epperson F., Tallman E.F., Quaid K.A., Farlow M.R., Saykin A.J., Ghetti B. (2016). [(11)C]PiB PET in Gerstmann-Sträussler-Scheinker disease. Am. J. Nucl. Med. Mol. Imaging.

[9] Baiardi S., Rizzi R., Capellari S., Bartoletti-Stella A., Zangrandi A., Gasparini F., Ghidoni E., Parchi P. (2020). Gerstmann-Sträussler-Scheinker disease (PRNP p.D202N) presenting with atypical parkinsonism. Neurol. Genet..

[10] Stephen C.D., de Gusmao C.M., Srinivasan S.R., Olsen A., Freua F., Kok F., Montes Garcia Barbosa R., Chen J.Y.H., Appleby B.S., Prior T., Frosch M.P., Schmahmann J.D. (2024). Gerstmann-Sträussler-Scheinker Disease Presenting As Late-Onset Slowly Progressive Spinocerebellar Ataxia, And Comparative Case Series With Neuropathology. Mov. Disord. Clin. Pract..

[11] Ono N., Suzuyama K., Minagawa H. (2024). Involvement of the nigrostriatal system in Gerstman-Sträussler-Scheinker disease with the PRNP-P102L mutation. J. Neurol. Sci..

[12] Appleby B.S., Shetty S., Elkasaby M. (2022). Genetic aspects of human prion diseases. Front. Neurol..

